# Training a Spinal Cord Injury Rehabilitation Team in Motivational Interviewing

**DOI:** 10.1155/2015/358151

**Published:** 2015-12-06

**Authors:** Pilar Lusilla-Palacios, Carmina Castellano-Tejedor

**Affiliations:** ^1^Department of Psychiatry, Vall d'Hebron University Hospital, CIBERSAM, Passeig de la Vall d'Hebron 119-129, 08035 Barcelona, Spain; ^2^Vall d'Hebron Research Institute, Passeig de la Vall d'Hebron 119-129, 08035 Barcelona, Spain; ^3^Autonomous University of Barcelona, Campus UAB, Bellaterra, 08193 Barcelona, Spain

## Abstract

*Background*. An acute spinal cord injury (ASCI) is a severe condition that requires extensive and very specialized management of both physical and psychological dimensions of injured patients.* Objective*. The aim of the part of the study reported here was twofold: (1) to describe burnout, empathy, and satisfaction at work of these professionals and (2) to explore whether a tailored program based on motivational interviewing (MI) techniques modifies and improves such features.* Methods*. This paper presents findings from an intervention study into a tailored training for professionals (*N* = 45) working in a spinal cord injury (SCI) unit from a general hospital. Rehabilitation professionals' empathy skills were measured with the Jefferson Scale of Physician Empathy (JSPE), burnout was measured with the Maslach Burnout Inventory (MBI), and additional numeric scales were used to assess the perceived job-related stress and perceived satisfaction with job.* Results*. Findings suggest that professionals are performing quite well and they refer to satisfactory empathy, satisfaction at work, and no signs of burnout or significant stress both before and after the training.* Conclusions*. No training effect was observed in the variables considered in the study. Some possible explanations for these results and future research directions are discussed in depth in this paper. The full protocol of this study is registered in ClinicalTrials.gov (identifier: NCT01889940).

## 1. Introduction

Health professionals working in an acute spinal cord injury (ASCI) unit have to face a challenging situation. On the one hand, patients with a recent traumatic spinal cord injury (SCI) are in a severe and very difficult moment of their lives and need constant and accurate care. On the other hand, the rehabilitation team has to deal with the psychological consequences of ASCI which reach beyond patients themselves as they affect patients' families as well as rehabilitation professionals [1]. Consequently, professionals must tackle a complex biomedical condition while simultaneously addressing the psychoemotional needs of patients and their families [2, 3].

Professional training in rehabilitation has traditionally focused on the biomedical aspects; thus, many rehabilitation professionals lack expertise in managing the psychological conditions that are extremely common in ASCIs [3]. Up to 47% of ASCI patients exhibit psychoemotional impact throughout the different stages of their hospitalization [4], with higher rates during the acute phase and at discharge [5]. Considering this high prevalence of psychological distress among ASCI patients, it is especially important to highlight that most of them consider that their emotional needs are not sufficiently addressed by their rehabilitation team, as some research has pointed out [6, 7]. In this sense, empathy is a core skill when communicating and interacting with patients. Empathy has been defined as the ability of understanding patients' feelings and concerns and it has been related to increased likelihood of patients' adherence to treatment [8]. Although the roles of empathy, collaborative care, and communication skills are emphasized as core competencies for new residents in physical medicine and rehabilitation [9], there are very few studies focused on developing a specific training in these issues and, as far as we know, any of these studies have been focused on managing ASCI patients [10]. Moreover, there is a lack of information about whether senior health professionals working with ASCI patients exhibit those competences or not in their interventions.

When patients have unrealistic expectations and medical staff has poor empathy skills, conflicts can arise, clinical outcomes can become compromised, and professionals can suffer from stress, job dissatisfaction, and/or increased symptoms of burnout as a consequence [11, 12]. Burnout is a highly prevalent syndrome described among health professionals as a consequence of daily dealing with high demanding severe patients and a lack of resources. It is characterized by emotional exhaustion, depersonalization, and lack of personal accomplishment at work [13]. Although no specific studies have been carried out with rehabilitation staff working with ASCI patients, there are reports showing that professionals working in other rehabilitation units suffer from emotional exhaustion and signs of depersonalization [14]. Related to these results, Lamothe and colleagues have found that those GPs with higher empathic concern and higher understanding of patients' perspectives exhibit lower levels of burnout [15]. Additionally, burnout has systematically appeared inversely related to career satisfaction among healthcare professionals [16]. Similarly, data indicate that teaching communication skills to physicians is related to greater satisfaction among professionals and reduced stress [17].

A major aspect is the type of training provided. Motivational interviewing (MI) is a patient-centered approach to enhancing patient-health professional collaboration through an emphatic listening among other communication skills [18]. MI can enhance a more compassionate and empathic approach to the patients' needs and emotions. That leads to a higher sense of mastery among health professionals and consequently to increased satisfaction and reduced job-related stress. Moreover, there are evidences of the efficacy and acceptance of training MI communication skills in health professionals [19].

To summarize, there are recent studies that point out the relevance of collaborative care interventions and incorporate MI in the initial care management of psychological distress in injured trauma survivors, although patients with ASCI are usually excluded [20]. As far as we know, there is no previous research in training rehabilitation professionals in psychoemotional aspects of ASCI patients and there are no references using MI for this purpose.

The present study is, to our knowledge, the first evaluation of an MI training involving the whole staff members of a high-specialized ASCI unit from a general hospital. The aim of the part of the study reported here was twofold: (1) to describe burnout, empathy, and satisfaction at work of these professionals and (2) to explore whether a tailored program based on motivational interviewing (MI) techniques modifies and improves such features. We hypothesized that health professionals participating in this tailored training would increase their self-perceived level of empathy. Additionally, whether the training might improve trainees' self-perceived satisfaction at work and decrease job-related stress and burnout will be explored.

## 2. Methods

### 2.1. Study Design

A quasiexperimental pre-post control design has been employed.

### 2.2. Participants

The whole rehabilitation staff working in the SCI unit was invited to participate in the study (*N* = 63) on a voluntary basis. This staff included nurses, assistant nurses, physiotherapists, assistant physiotherapists, fitness monitors, rehabilitation physicians, occupational therapists, social workers, and hospital attendants. Both genders were included and no age limits were set up. CME credits were provided after the training.

### 2.3. Procedure

Informative sessions about the project to the rehabilitation staff were scheduled after the approval from the different team leaders involved (physiotherapy, hospital attendants, and nursing staff). In these sessions, carried out by the main researcher of the study, voluntary participation was asked and informed consent was collected.

The initial assessment (baseline, months 1–12) included pretraining measures for empathy, burnout and stress, and satisfaction at work. Demographic data was collected too. All assessments were carried out by a researcher with a doctorate degree in health psychology.

After the initial assessments, and prior to the training, separate focus groups for the rehabilitation team as well as patients and their families were formed to determine needs for and concerns over ASCI rehabilitation (month 13-14). A focus group was conducted with non-ASCI patients (patients who had an ASCI ≥1 year ago and are now considered as chronic patients) and their main caregivers. This focus group provided qualitative information about the first period of ASCI and covered areas such as psychoemotional needs, information delivery of health issues, and the more helpful staff attitude/skills. The rehabilitation team focus groups covered the following areas: (a) delivering bad news to patients and their families, (b) teamwork and organizational affairs, (c) handling of difficult patients, (d) managing psychoemotional reactions, (e) helping patients to accept their injury, and (f) dealing with distressed and/or demanding families.

Training was designed* ad hoc*, according to the relevant issues identified through focus groups and following the principles of MI framework [18]. It also included theoretical and practical exercises on early detection of psychological distress, how to show empathy, communication skills, management of patients' difficult reactions, family interventions, and teamwork alternatives. Moreover videos with real patients and role-playing were used along the training. Standard MI techniques for improving empathy skills and communication styles were employed [18]. The focus groups and the sessions were led by two expert trainers, members of the International MI Network of Trainers, and the previously mentioned researcher psychologist. This training was provided for 12 hours (two-day training) (months 15–17).

Immediately after the training, coaching was delivered on demand, individually, or in small groups, in sessions of 50 to 60 minutes during a six-month period (months 18–23). The aim was to strengthen previously acquired skills and discuss practical cases. The coaching also employed MI techniques.

Lastly, once the coaching period ended, a voluntary 2-hour reviewing session was offered to revisit and consolidate concepts (month 24). This session was also led by an expert trainer in MI and a researcher psychologist.

At the end of all these actions (training, coaching, and reviewing session) professionals were assessed again (posttraining measures, months 25–30). It was a three-year protocol [10].

### 2.4. Assessment Tools

Rehabilitation professionals' empathy skills were measured with the* Jefferson Scale of Physician Empathy (JSPE)* [21] created by Hojat et al. [22, 23] to assess empathy in the context of medical education and patient care. It encompasses 20 items answered on a 7-point Likert scale. General scores range from 20 to 140, with higher scores indicating a more empathic orientation toward patient care. The JSPE relies on the definition of empathy in the context of patient care as “a predominantly cognitive attribute that involves an understanding of patient's experiences, concerns and perspectives, combined with a capacity to communicate this understanding and an intention to help” [8]. The JSPE also provides scores on three empathy dimensions: “taking perspective”, “compassionate care”, and professional ability “to stand in patient's shoes” [21]. In our sample, Cronbach's Alpha was 0.76 at baseline and 0.60 after intervention.

Rehabilitation professionals' level of burnout was measured with the* Maslach Burnout Inventory (MBI)*, designed by Maslach et al. [13]. The MBI contains 22 items answered on a 7-point Likert scale that measure three dimensions of burnout among health professionals: (1) emotional exhaustion, (2) depersonalization, and (3) lack of personal accomplishment. To consider the existence of burnout syndrome, high scores in emotional exhaustion and depersonalization and low scores on personal accomplishment are needed. It is widely used in different medical settings in various countries [24]. Cronbach's Alpha was the following: for emotional exhaustion, 0.74 and 0.86 before and after intervention; for depersonalization, 0.36 and 0.32; and for the dimension lack of personal accomplishment 0.77 and 0.70, respectively.

An additional numeric scale to assess the perceived* job-related stress* was used. This consisted of a 5-point numeric scale from* 1—little stressful*—to* 5*—*very stressful*. Similarly,* perceived satisfaction with job* was evaluated with a numeric scale too, designed* ad hoc* as a 5-point numeric scale ranging from* 1*—*unsatisfactory*—to* 5*—*very satisfactory.*

### 2.5. Statistical Analyses

Data were analyzed using the statistical package for social sciences (SPSS). To describe the sample, descriptive analyses were performed (frequencies, central tendency, and dispersion measures). The main variables were also subject to descriptive, correlational, and comparative analysis according to the intervention period group (before training versus after training, within subject design). Primary analysis (comparison of pre-post) entailed either parametric (*t*-test) or nonparametric (Mann-Whitney *U*) tests, depending on the sample data distribution. Significance level was set as *p* < 0.05.

## 3. Results

From an initial pool of 63 professionals working in the SCI unit, a total of 45 professionals were assessed (before/after training). This represented 71.4% of the rehabilitation staff at the moment of the study. Missing sample in posttraining assessments was due to retirement (*n* = 1), sick or maternity leaves (*n* = 5), change of service (*n* = 7), or not wanting to answer again the questionnaires (*n* = 5).

Demographic and job-related characteristics of the sample are displayed in Table 1.

Most professionals were females (75.6%), married (44.4%), and working as nurses (31%). The average length of time of working with ASCI patients was almost 19 years, which means that this is a very experienced and specialized staff.

No significant differences were found before/after intervention with regard to sociodemographics (gender, age, and marital status) and the basic job-related variables considered in this study (profession and time working in the field), in relation to burnout, empathy and stress, and job satisfaction, either at baseline or after intervention.

Descriptive results regarding pre/posttraining measures are displayed in Table 2.

As it can be observed, before the training, a medium level of empathy was obtained. Concerning burnout, a low level of emotional exhaustion (EE) and depersonalization (DP) was shown. Personal accomplishment (PA) showed high scores. With regard to job-related self-perceived stress, the staff exhibited a medium level of stress and their self-perceived job satisfaction was quite high.

After intervention, scores for the whole sample kept the general sense of preintervention outcomes. Thus, medium levels of empathy, low levels of EE, DP, and high levels of PA were displayed. Similarly, job-related stress was medium and self-perceived satisfaction with work was high.

On the whole, no significant differences were observed between pre/posttraining measures for any of the variables assessed. The only observed difference was in women, who showed significantly higher preintervention scores compared to men in EE (M = 15.85, SD = 6.63 versus M = 10.27, SD = 6.40; *t* = −2.446_(43)_, *p* = 0.019, 95% CI −10.181–−0.978) and in “ability to stand in patients' shoes” (M = 14.38, SD = 2.83 versus M = 12.09, SD = 3.53; *t* = −2.191_(43)_, *p* = 0.034, 95% CI −4.40–−0.182).

## 4. Discussion

The goal of this study was to determine whether a tailored training in MI skills increases empathic attitudes and job satisfaction and decreases burnout and job-related stress in staff working in a SCI Unit.

Prior to the training, the staff working in the SCI unit displayed a high average of almost 19 years working in this specific field. Therefore, one could infer a high specialization and broad experience working with these patients and their specific characteristics. In this sense, they showed low levels of job-related stress and medium-to-high levels of satisfaction with doing their job. Similarly, there are no data referring to a possible burnout syndrome since all mean scores regarding EE and DP are low (≤16 and ≤8, resp.), and PA reaches high scores (≥37, high). Besides, self-perceived empathy has been rated as medium-to-high (M = 113.71, SD = 12.84, in a possible range of 20–140 points). Thus, data seems to indicate that professionals are performing their jobs at an acceptable level and have certain degree of self-perceived competence while performing their regular activity with ASCI patients. A large experience working with ASCI patients may explain these positive results. A highly demanding job requires high specialization and a certain degree of stress. However, after more than a decade of working with ASCI patients, professionals referred to experience satisfaction at work and expressed being able to display empathy to their patients in a quite satisfactory manner. This may also explain the low level of burnout found. Burnout syndrome is a progressive psychological weakness caused by a misbalance of the task demands and the personal resources to cope, and it is typically defined as high scores in EE and DP and low scores in PA [13]. There is evidence that burnout is highly prevalent among health professionals and rates rank between 30 and 50% depending of the study [25]. Nevertheless, we have not found such symptoms in our sample. Although burnout is commonly linked to the assistance of severe patients, some protective factors have been described. For example, team support and cohesion, the organization commitment to safeguard the health of their employees, and the experience of professionals themselves.

After the training all mean scores remained in the same previous satisfactory parameters, with no statistical differences. The only difference was observed in women who showed significantly higher preintervention scores in EE and “ability to stand in patients' shoes” (JSPE dimension) compared to men. However, this difference disappeared after the training. Perhaps, the training has helped to improve stress management skills and coping with difficult situations among professionals, blending differences linked to gender. Besides, job satisfaction, which has been revealed as high in our sample, is commonly related to higher degree of commitment, motivation towards work, and lower degree of job-related stress and burnout [26].

No differences were found regarding professional category or years working in the field and empathy, job satisfaction, job-related stress, or burnout. However, we cannot conclude that such variables are not related since our sample was relatively small and it could have limited statistical power. Literature is inconsistent on gender's relationship to empathy and the same happens with educational level [27–29]. However, some studies showed that empathy with patients seems to differ among medical students in various years of their education and thus the amount of empathy with patient reduces with increase in their age and educational level [23, 27–29].

Research shows that training programs in communications skills are more effective when delivered in small groups, when accurate feedback is given, and when coaching is provided after the initial training [28, 29]. This has also been shown in the acquisition of MI skills [18]. Moreover, MI has shown to increase the empathy level of health professionals and decrease their burnout [30]. However, this has not been proved in our study, possibly due to the satisfactory baseline scores on such features. There are several studies that point out that basic workshops do not produce sufficient competence in MI and that it takes much more practice in real-life situations (rather than role-playing). This implies that trainees may need longer-term continuing supervision and support than previously recognized [31].

There are several reasons for not observing significant differences after the training. First of all, it is possible that professionals could overestimate their empathy and we do not have reports from patients who could actually evaluate more accurately the professionals' empathy. Secondly, we have not included specific measures to assess the professionals' real performance when facing patients and how they integrate the MI skills into their daily practice. Therefore, it is possible that some improvements have been missed. We believe it could be of relevance to include qualitative data regarding self-perceived improvements in their daily practice. Similarly, it has been assumed that empathy will be displayed throughout the specific communication skills trained. However, empathy is a broader construct and to collect other parameters could be of interest (e.g., attitude towards the patient and showing genuine interest). Finally, a longer-term training and supervised practice could yield better results. Coaching and reviewing sessions were not demanded for all professionals and, as some research has demonstrated, postworkshop inputs such as feedback and coaching assessing performance after training are usually needed to sustain training gains over time [32].

Despite these limitations, this is the first study that designs and implements a tailored specific training for a whole SCI unit staff. On the one hand, it is a major asset since all ASCI units are constituted by these professional categories and they are used to working together. Therefore, synergies and common agreements on basic management of psychosocial aspects of patients are necessary. On the other hand, representativeness of results can be limited due to the heterogeneity of professionals with different backgrounds and capabilities.

### 4.1. Clinical Implications and Conclusions

Our results show that the staff experience high levels of job satisfaction and satisfactory empathy was found. Similarly, burnout and job-related stress were quite low and no specific risk profiles were identified among the staff. Moreover, the professionals' ratings of the training were very satisfactory and as they refer to the researchers, they felt an increased sense of mastery, self-confidence, and cohesion in the SCI staff. Despite potentialities of MI training in healthcare settings, significant improvements were not observed in any of the assessed variables in our sample. We can explain such results due to the good starting point in baseline measures. However, there are still several aspects that must be considered. When designing a training intervention for SCI unit staff, it is crucial to assess not only the professionals' perspective of their performance but that of their patients (e.g., to rate more accurately empathy). Additionally, this training should be delivered in small groups where immediate and personalized feedback can be provided. It is also important to assess specific performance of such professionals in real situations to detect possible areas of improvement and to design regular boosting sessions/coaching sessions to review and practice contents. Similarly, it is important to consider measuring not only burnout, empathy, job-related stress, or satisfaction, but other variables that can be affected by this specific MI training (e.g., mastery, self-efficacy, and confidence). Finally, we think it is vital to extend this methodology to other SCI units and sectors of healthcare to replicate our study and compare results.

## Figures and Tables

**Table 1:** Demographic and job-related characteristics of the sample (*N* = 45).

	*n*	%
Gender		
Male	11	24.4
Female	34	75.6
Marital status		
Single	10	22.2
Married	20	44.4
Steady partner	6	13.3
Divorced	8	17.8
Widow(er)	1	2.2
Profession		
Nurse	14	31
Assistant nursing	9	20
Physiotherapist	6	13.3
Physiotherapist assistant	1	2.2
Fitness monitor	2	4.4
Rehabilitation physician	3	6.6
Occupational therapist	1	2.2
Social worker	3	6.7
Hospital attendants	6	13.3

	Mean (SD)	Range

Age at assessment (in years)^†^	45.2 (10.5)	28–62
Time working in the field (in years)	18.4 (10.6)	2–38

^†^Three missing values.

**Table 2:** Descriptive results (*N* = 90).

Factor	Pretraining results (*n* = 45)		Posttraining results (*n* = 45)		Scores' interpretation
	Mean (SD)	Range	Mean (SD)	Range	
JSPE: empathy total scores	113.71 (12.84)	77–134	114.67 (10.15)	92–138	20–140 (↑ scores, ↑ empathy)
JSPE: taking perspective (10 items)	58.75 (7.31)	42–70	59.42 (6.70)	40–69	10–70 (↑ scores, ↑ empathy)
JSPE: compassionate care (7 items)	41.13 (6.64)	20–49	41.95 (5.91)	24–49	7–49 (↑ scores, ↑ empathy)
JSPE: ability to stand in patients' shoes (3 items)	13.82 (3.14)	6–20	13.29 (3.19)	8–21	3–21 (↑ scores, ↑ empathy)
MBI: emotional exhaustion	14.49 (6.94)	1–34	15.40 (8.14)	2–46	≤16 low, 17–26 medium, and ≥27 high
MBI: depersonalization	3.33 (2.81)	0–11	3.27 (2.81)	0–11	≤8 low, 9–13 medium, and ≥14 high
MBI: personal accomplishment	39.73 (5.95)	23–48	38.80 (5.53)	24–47	≤30 low, 31–36 medium, and ≥37 high
NS: self-perceived stress at work	3.31 (0.79)	1–5	3.18 (1.03)	1–5	1–5 (↑ scores, ↑ stress)
NS: self-perceived job satisfaction	4.20 (0.66)	3–5	4.22 (0.70)	3–5	1–5 (↑ scores, ↑ satisfaction)

JSPE: Jefferson Scale for Physician Empathy; MBI: Maslach Burnout Inventory; NS: numeric scale.

## Abbreviations

ASCI: Acute spinal cord injury
JSPE: Jefferson Scale of Physician Empathy
MBI: Maslach Burnout Inventory
MI: Motivational interview(ing)
SCI: Spinal cord injury.
